# Author Correction: From short-term uncertainties to long-term certainties in the future evolution of the Antarctic Ice Sheet

**DOI:** 10.1038/s41467-026-71978-9

**Published:** 2026-04-13

**Authors:** Violaine Coulon, Ann Kristin Klose, Tamsin Edwards, Fiona Turner, Frank Pattyn, Ricarda Winkelmann

**Affiliations:** 1 Université libre de Bruxelles (ULB), Laboratoire de Glaciologie, Brussels, Belgium; 2 Potsdam Institute for Climate Impact Research (PIK), Member of the Leibniz Association, Potsdam, Germany; 3 Department of Physics and Astronomy, University of Potsdam, Potsdam, Germany; 4 King’s College London, Department of Geography, London, UK; 5 Integrative Earth System Science, Max Planck Institute of Geoanthropology, Jena, Germany

**Keywords:** Cryospheric science, Climate change

Correction to: *Nature Communications* 10.1038/s41467-025-66178-w, published online 5 December 2025

In the initially published version of this article, in Table 2, the ranges of Mplume and Mquad for the P5 parameter now reading “1–10 × 10⁻⁴” were originally listed as “0.1–10 × 10⁻⁴.” The table is updated in the HTML and PDF versions of the article.

