# Supplementary material for: From short-term uncertainties to long-term certainties in the future evolution of the Antarctic Ice Sheet
Source: Nat Commun. 2025 Dec 5;16:10385. doi: 10.1038/s41467-025-66178-w (PMC12680641; doi:10.1038/s41467-025-66178-w)
Supplement: Supplementary file 1 — Supplementary Information [file 41467_2025_66178_MOESM1_ESM.pdf]

Supplementary Information

From short-term uncertainties to long-term certainties in the future evolution of the Antarctic Ice Sheet

Violaine Coulon[1*†], Ann Kristin Klose[2,3*†], Tamsin Edwards[4], Fiona Turner[4], Frank Pattyn[1], Ricarda Winkelmann[2,3,5]

[1]Université libre de Bruxelles (ULB), Laboratoire de Glaciologie, Brussels, Belgium.
[2]Potsdam Institute for Climate Impact Research (PIK), Member of the Leibniz Association, P.O. Box 6012 03, 14412 Potsdam, Germany.
[3]Department of Physics and Astronomy, University of Potsdam, Potsdam, Germany.
[4]King's College London, Department of Geography, London, UK.
[5]Integrative Earth System Science, Max Planck Institute of Geoanthropology, 07745 Jena, Germany.

*Corresponding author(s). E-mail(s): violaine.coulon@ulb.be; annkristin.klose@pik-potsdam.de;
†These authors contributed equally to this work.

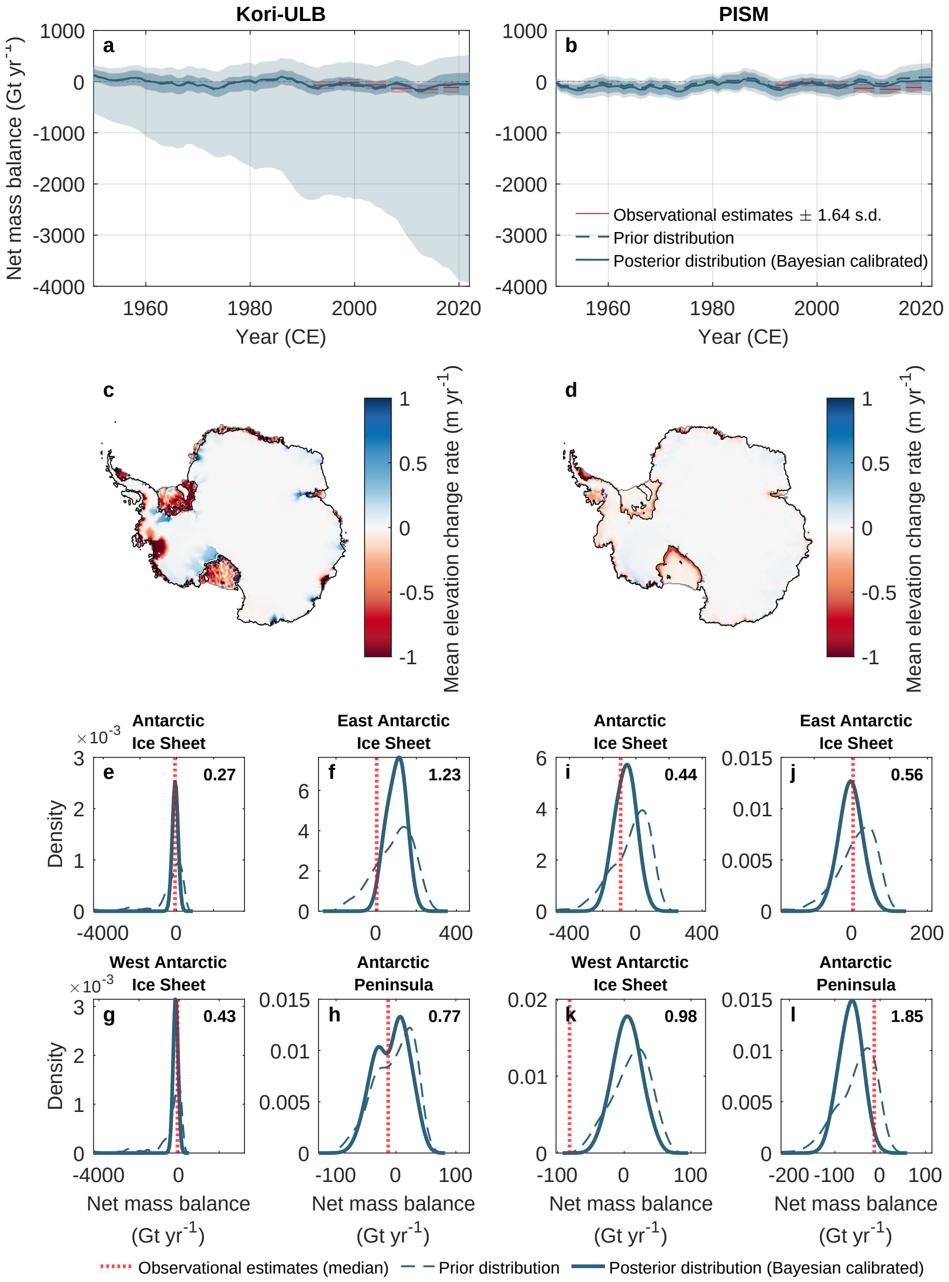

Fig. S1 Influence of calibration in Bayesian framework and calibrated trends over historical period. a and **b** Evolution of the Antarctic Ice Sheet net mass balance in response to historical changes in Antarctic climate derived from NorESM1-M as determined by the ice-sheet models Kori-ULB and PISM, compared to observations (IMBIE[1]; see Supplementary Table 2, with mean and uncertainty given as pink solid lines and shading, respectively). The ensemble prior distributions (medians and 5%–95% probability intervals) are indicated by dashed lines and pale blue shaded areas, while solid lines and dark blue shaded areas show the posterior distributions (Bayesian-calibrated medians and 5%–95% probability intervals). **c** and **d** Bayesian-calibrated mean rates of ice-elevation change. For comparison, the observed mean elevation change rate[2] is shown in Supplementary Figure 8. **e**–**l** Comparison of modeled and observed Antarctic mass balance (1992-2020 average) at the continental scale and for different Antarctic ice-sheet regions for the ice-sheet models Kori-ULB and PISM. Shown are the prior (uncalibrated) and posterior (Bayesian calibrated) probability density functions of the 1992-2020 mean net mass balance as the dashed and solid blue lines, respectively. Observational estimates from IMBIE[1] are indicated by red dashed lines. Normalized Continuous Ranked Probability Score (CRPS; see **Methods**) values are shown in the top right corner, quantifying how the calibration influences the ice-sheet model–observation agreement. The scores shown are normalized by the CRPS of the prior (uncalibrated) distribution, so a normalized CRPS below 1 indicates that Bayesian calibration has improved the agreement with observations.

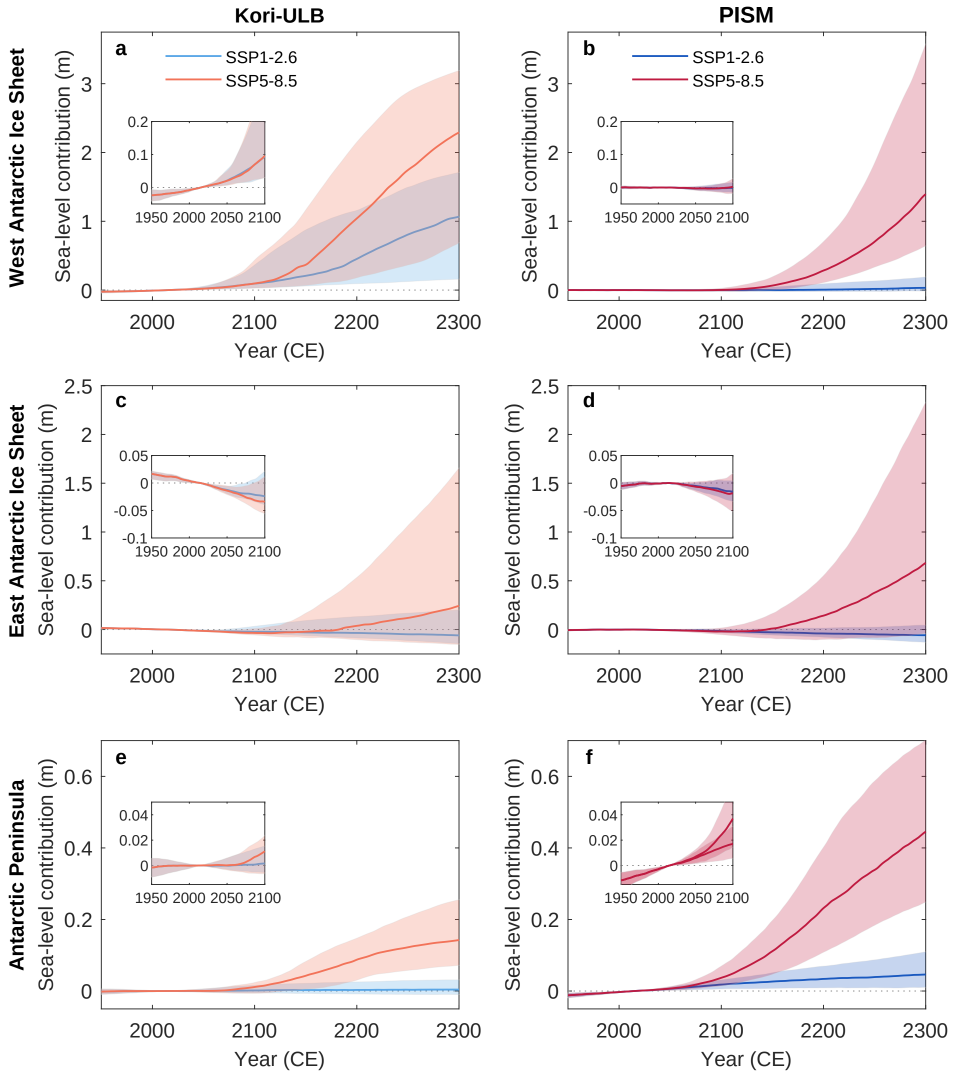

Fig. S2 Future sea-level contribution from different Antarctic Ice Sheet regions through 2300 (in meters sea-level equivalent) in response to changes in Antarctic climate projected by four CMIP6 GCMs under emission pathways SSP1-2.6 (blue) and SSP5-8.5 (red). Solid lines and shaded areas show the medians and 5–95% probability intervals of the calibrated probabilistic sea-level projections by the ice-sheet models Kori-ULB (left column, lighter colors, **a**, **c** and **e**) and PISM (right column, darker colors, **b**, **d** and **f**). The future sea-level contribution from West Antarctica (**a** and **b**), East Antarctica (**c** and **d**), and the Antarctic Peninsula (**e** and **f**) is shown.

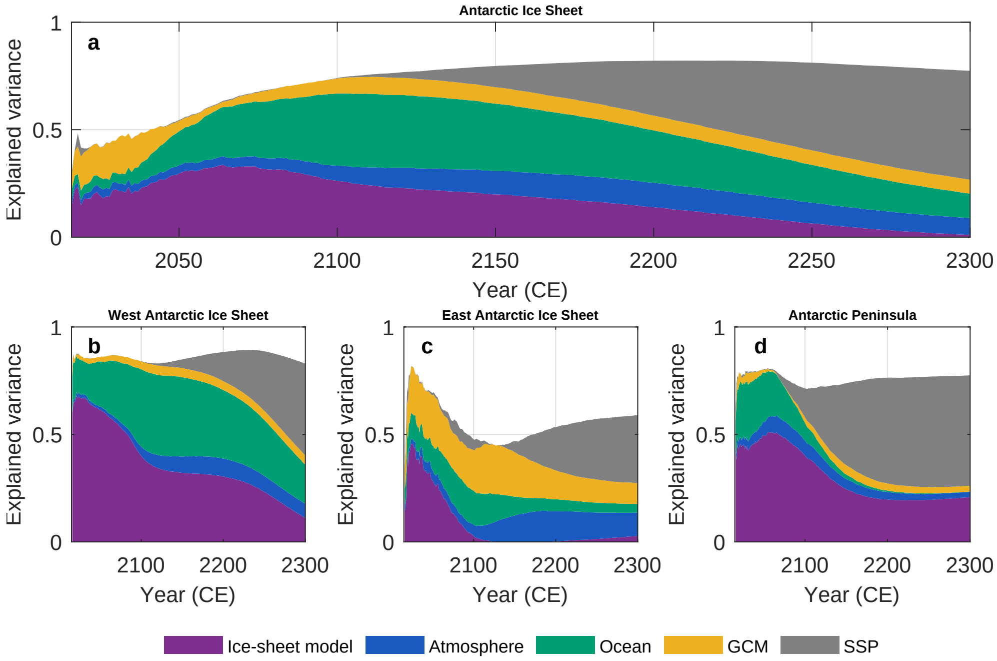

Fig. S3 Sources of uncertainty in the future sea-level contribution from the Antarctic Ice Sheet through 2300. Explained variance (i.e., the fraction of total variance) in the Antarctic ice-sheet contribution to global mean sea level attributed to main effects of the ice-sheet model (purple), atmosphere-related parameters (blue), ocean-related parameters (green), CMIP6 GCMs (yellow), and the emission scenario (SSP, grey), based on ANOVA for the combined Kori-ULB and PISM ensembles. Each colored area represents the fraction of total ensemble variance explained by a given source of uncertainty or interaction over time. Fractions from all individual atmospheric- and ocean-related parameters are aggregated into their respective categories. The white space above the stacked areas represents variance contributions from two-way and higher-order interaction terms that are not shown here, as well as residual unexplained variance. Results are shown for the Antarctic Ice Sheet (**a**), the West Antarctic Ice Sheet (**b**), the East Antarctic Ice Sheet (**c**), and the Antarctic Peninsula (**d**).

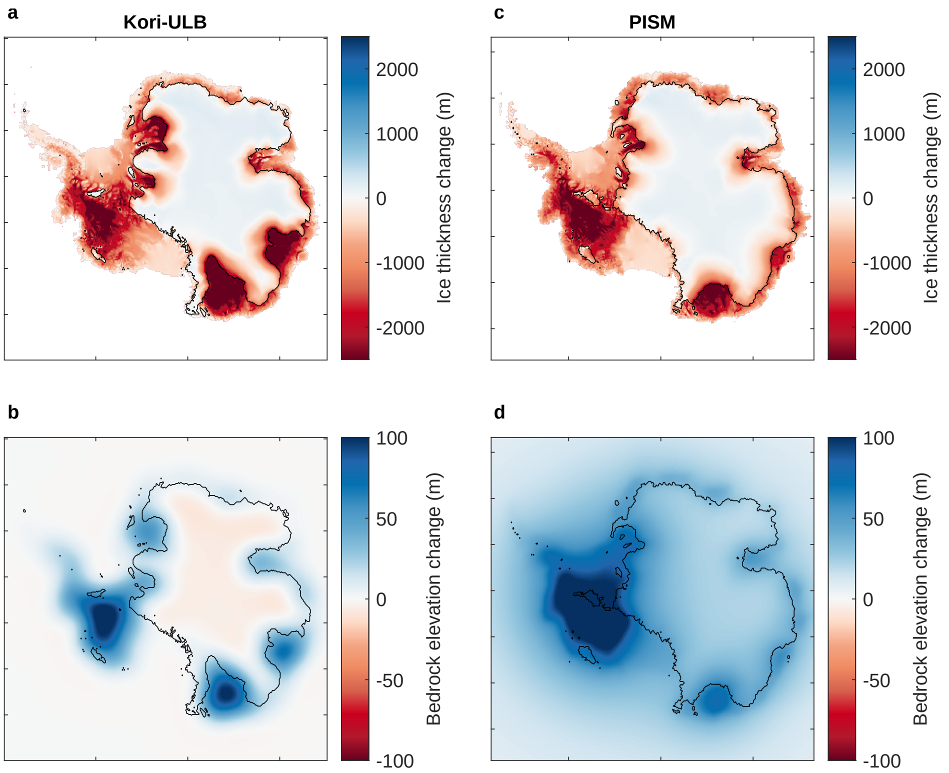

Fig. S4 Comparison of isostatic responses to changes in ice loading between the ice-sheet models Kori-ULB and PISM. (**a**–**b**) Ice thickness change by year 3000 projected by Kori-ULB (**a**) and PISM (**b**) under the SSP5-8.5 emission pathway. (**c**–**d**) Corresponding bedrock elevation change by year 3000 as determined by Kori-ULB (**c**) and PISM (**d**). Black lines show the grounding line position. Shown is one simulation selected from each ice-sheet model ensemble.

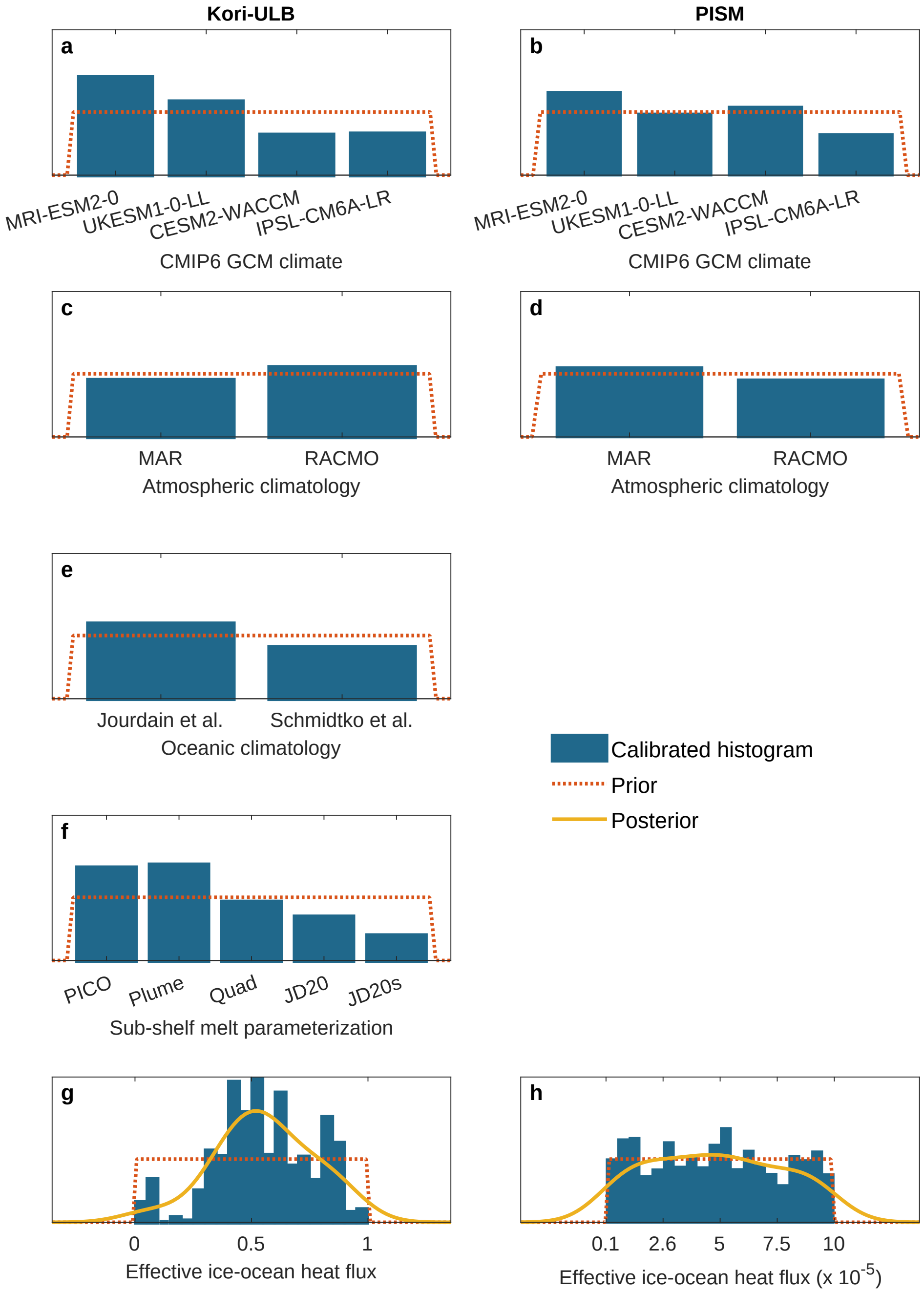

Fig. S5 Posterior probability distribution of each ice-sheet model's parameter space. Posterior density histograms (in blue) and probability distribution functions (approximated using kernel density estimation, as solid yellow line) are shown for all parameters in the ensembles of the ice-sheet models Kori-ULB (left column; **a**, **c**, **e**, **f**, **g**) and PISM (right column; **b**, **d**, **h**), except for the discrete inputs (CMIP6 GCM climate, atmospheric and oceanic present-day climatology, and, for the Kori-ULB ensemble, sub-shelf melt parameterization). In addition, the prior distributions are given as dashed red lines. For Kori-ULB, the uncertainty range of the effective ice-ocean heat flux depends on the applied sub-shelf melt parameterization. The range shown in **g**, [0,1], corresponds to the full sampling interval defined by the Latin Hypercube sampling.

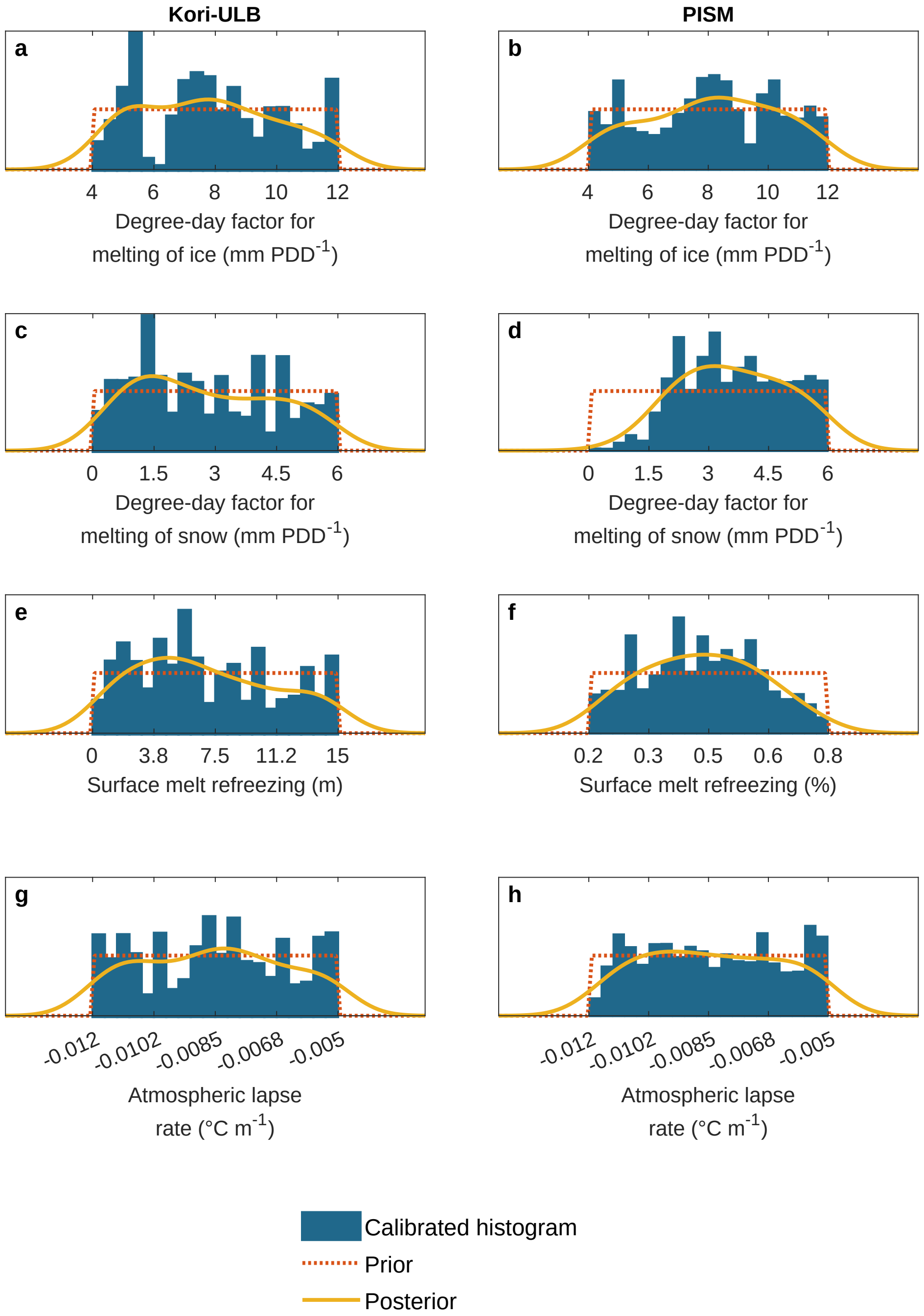

Fig. S6 Posterior probability distribution of each ice-sheet model's parameter space. Posterior density histograms (in blue) and probability distribution functions (approximated using kernel density estimation, as solid yellow line) are shown for all parameters in the ensembles of the ice-sheet models Kori-ULB (left column; **a**, **c**, **e**, **g**) and PISM (right column; **b**, **d**, **f**, **h**). In addition, the prior distributions are given as dashed red lines.

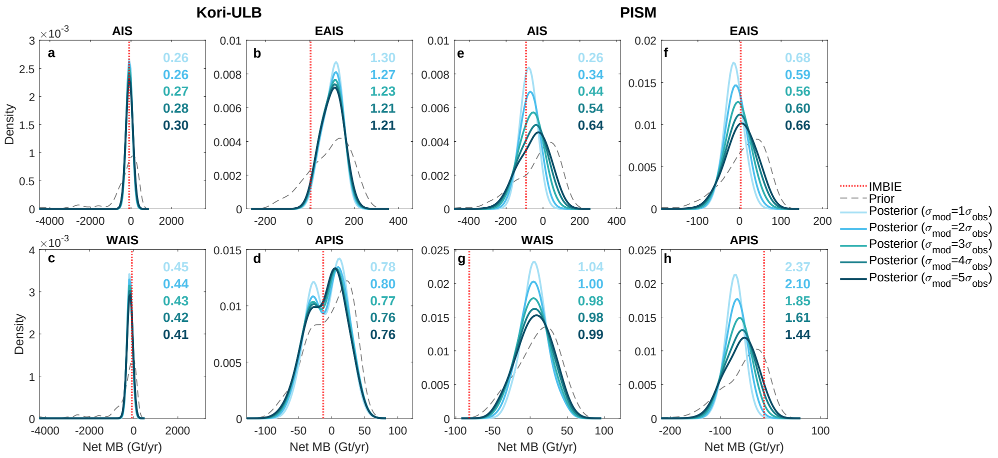

Fig. S7 Influence of the choice of the structural error on the comparison between modeled and observed Antarctic mass balance (1992-2020 average) at the continental scale and for different Antarctic Ice Sheet regions for Kori-ULB (a–d) and PISM (e–h). Each subplot shows the probability density functions of the 1992-2020 mean mass balance from the ensemble: the dashed grey lines show the prior (uncalibrated) distributions while the solid blue lines show the posterior (Bayesian calibrated) distributions obtained with different values of the structural error (1, 2, 3, 4, and 5). Observational estimates from IMBIE[1] are indicated by red dashed lines. Normalized Continuous Ranked Probability Score (CRPS; see **Methods**) values are shown in the top right corner of each subplot, quantifying how calibration influences model–observation agreement. Lower CRPS values indicate better agreement. The scores shown are normalized by the CRPS of the prior (uncalibrated) distribution, so a normalized CRPS below 1 indicates that Bayesian calibration has improved the agreement with observations

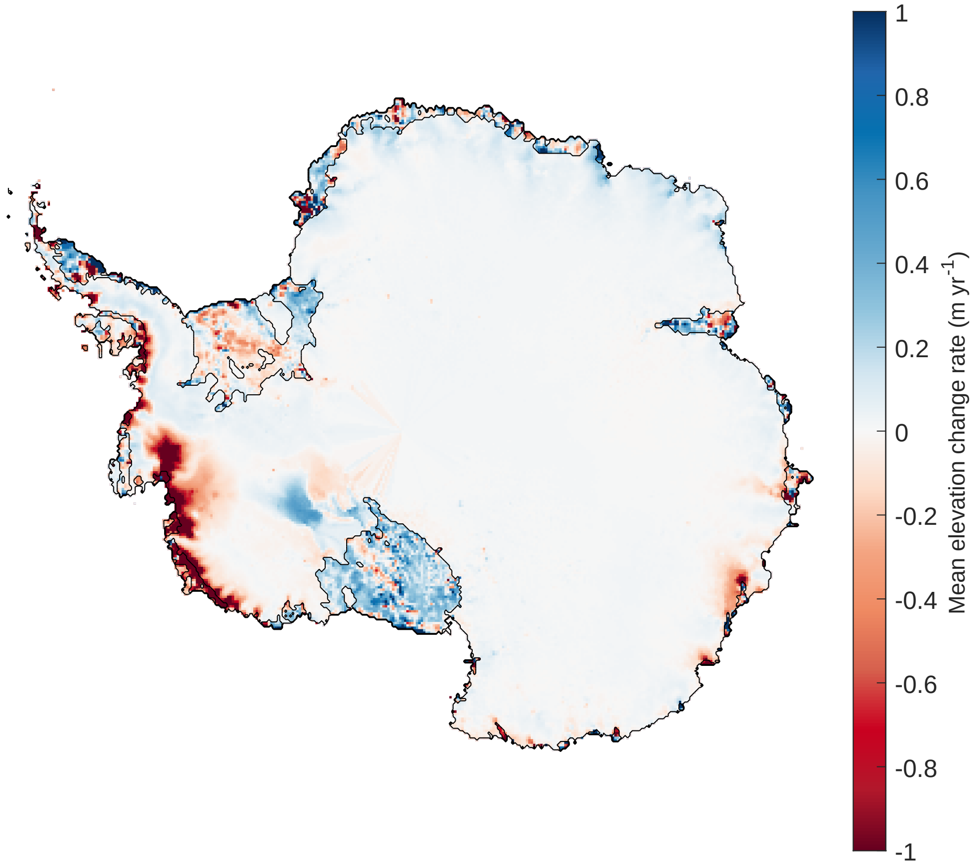

Fig. S8 Observed Antarctic mass change between 2003 and 2019. Mean elevation-change rate is inferred from ICESat satellite altimetry [2]

.

Table S1 Comparison of ice-sheet models with respect to the model physics, numerical methods, and initialization approaches.

	Kori-ULB	PISM
Stress balance	SSA+SIA (Hybrid)	SSA+SIA (Hybrid)
Sliding law	Weertman law	Power law with Mohr-Coulomb [3],
	$m = 3$	$q = 0.5$ and $q = 0.75$ (see **Methods**)
Calving	Crevasse-Depth [4,5]	Eigencalving [6], Thickness calving
Bed deformation	ELRA [7,8]	Lingle-Clark [9,10]
Numerical method	Finite difference	Finite difference
Initialization approach	Transient inverse simulation [11,12]	Spin-up
Grounding-line migration	Flux condition [11,13,14]	Subgrid interpolation

Table S2 Observed net mass balance of the Antarctic ice sheet over the past decades from the Ice Sheet Mass Balance Inter-comparison Exercise (IMBIE [1]). These estimates are used as observational constraints for the Bayesian calibrations of the ice-sheet model ensembles.

Time period	Value	Uncertainty	Unit
1992-1996	-70	40	$\mathrm{Gt\,yr^{-1}}$
1997-2001	-19	39	$\mathrm{Gt\,yr^{-1}}$
2002-2006	-62	41	$\mathrm{Gt\,yr^{-1}}$
2007-2011	-130	45	$\mathrm{Gt\,yr^{-1}}$
2012-2016	-150	43	$\mathrm{Gt\,yr^{-1}}$
2017-2020	-115	55	$\mathrm{Gt\,yr^{-1}}$

Table S3 Projected sea-level contribution from the Antarctic Ice Sheet on different timescales, depending on the choice of structural error.

	Kori-ULB				PISM			
	2100	2300	2500	3000	2100	2300	2500	3000
**SSP1-2.6**								
Prior	0.09	1.01	1.55	2.01	-0.02	-0.01	0.09	0.88
	[-0.11,1.22]	[-0.33,2.30]	[-0.54,3.05]	[-0.96,4.30]	[-0.06,0.07]	[-0.16,0.34]	[-0.22,1.04]	[-0.15,5.09]
Posterior,$\sigma_i^{mod} = 2\sigma_i^{obs}$	0.09	1.00	1.64	2.03	0.01	0.03	0.09	0.78
	[0.02,0.33]	[0.15,1.73]	[0.58,2.31]	[0.91,3.40]	[-0.02,0.04]	[-0.08,0.27]	[-0.11,1.00]	[0.02,6.02]
Posterior,$\sigma_i^{mod} = 3\sigma_i^{obs}$	0.09	1.10	1.65	2.04	0.00	0.03	0.10	0.81
	[0.01,0.33]	[0.07,1.74]	[0.41,2.31]	[0.85,3.41]	[-0.03,0.04]	[-0.09,0.30]	[-0.14,1.00]	[0.02,5.28]
Posterior,$\sigma_i^{mod} = 4\sigma_i^{obs}$	0.08	1.06	1.65	2.04	0.00	0.02	0.11	0.83
	[-0.01,0.33]	[0.07,1.75]	[0.11,2.43]	[0.80,3.41]	[-0.04,0.05]	[-0.13,0.30]	[-0.16,1.00]	[-0.07,5.25]
**SSP5-8.5**								
Prior	0.09	2.94	4.65	7.74	-0.02	2.26	6.75	12.46
	[-0.13,1.27]	[0.16,5.10]	[1.13,8.75]	[1.91,18.28]	[-0.10,0.16]	[0.47,6.18]	[2.47,13.28]	[7.02,25.84]
Posterior,$\sigma_i^{\mathrm{mod}} = 2\sigma_i^{\mathrm{obs}}$	0.08	2.69	4.04	6.14	0.03	2.85	7.87	13.70
	[0,0.37]	[0.73,5.04]	[1.44,8.38]	[1.88,14.62]	[-0.03,0.10]	[1.14,5.94]	[3.56,14.03]	[8.03,27.50]
Posterior,$\sigma_i^{mod} = 3\sigma_i^{obs}$	0.08	2.67	4.04	6.14	0.02	2.73	7.74	13.57
	[-0.01,0.37]	[0.73,5.09]	[1.44,8.42]	[1.88,17.53]	[-0.05,0.12]	[1.00,5.95]	[3.39,13.14]	[7.81,25.85]
Posterior,$\sigma_i^{mod} = 4\sigma_i^{obs}$	0.08	2.69	4.09	6.32	0.01	2.67	7.43	13.55
	[-0.01,0.37]	[0.71,5.09]	[1.60,8.62]	[2.22,17.53]	[-0.06,0.12]	[0.80,6.00]	[3.24,13.05]	[7.50,25.84]

Ice sheet changes (in meters sea-level equivalent) on the continental scale under emission pathways SSP1-2.6 and SSP5-8.5 as determined by the ice-sheet models Kori-ULB and PISM. Given are the medians and [5%–95%] probability intervals, projected by 2100 and 2300 next to the committed ice loss by 2500 and 3000, compared to 2015, for different choices of the structural error.

References

[1] Otosaka, I.N., Shepherd, A., Ivins, E.R., Schlegel, N.-J., Amory, C., Broeke, M.R., Horwath, M., Joughin, I., King, M.D., Krinner, G., Nowicki, S., Payne, A.J., Rignot, E., Scambos, T., Simon, K.M., Smith, B.E., Sørensen, L.S., Velicogna, I., Whitehouse, P.L., A, G., Agosta, C., Ahlstrøm, A.P., Blazquez, A., Colgan, W., Engdahl, M.E., Fettweis, X., Forsberg, R., Gallée, H., Gardner, A., Gilbert, L., Gourmelen, N., Groh, A., Gunter, B.C., Harig, C., Helm, V., Khan, S.A., Kittel, C., Konrad, H., Langen, P.L., Lecavalier, B.S., Liang, C.-C., Loomis, B.D., McMillan, M., Melini, D., Mernild, S.H., Mottram, R., Mouginot, J., Nilsson, J., Noël, B., Pattle, M.E., Peltier, W.R., Pie, N., Roca, M., Sasgen, I., Save, H.V., Seo, K.-W., Scheuchl, B., Schrama, E.J.O., Schröder, L., Simonsen, S.B., Slater, T., Spada, G., Sutterley, T.C., Vishwakarma, B.D., Wessem, J.M., Wiese, D., Wal, W., Wouters, B.: Mass balance of the Greenland and Antarctic ice sheets from 1992 to 2020. Earth System Science Data **15**(4), 1597–1616 (2023) https://doi.org/10.5194/essd-15-1597-2023

[2] Smith, B., Fricker, H.A., Gardner, A.S., Medley, B., Nilsson, J., Paolo, F.S., Holschuh, N., Adusumilli, S., Brunt, K., Csatho, B., Harbeck, K., Markus, T., Neumann, T., Siegfried, M.R., Zwally, H.J.: Pervasive ice sheet mass loss reflects competing ocean and atmosphere processes. Science **368**(6496), 1239–1242 (2020) https://doi.org/10.1126/science.aaz5845

[3] Schoof, C., Hindmarsh, R.C.A.: Thin-Film Flows with Wall Slip: An Asymptotic Analysis of Higher Order Glacier Flow Models. The Quarterly Journal of Mechanics and Applied Mathematics **63**(1), 73–114 (2010) https://doi.org/10.1093/qjmam/hbp025

[4] DeConto, R.M., Pollard, D.: Contribution of Antarctica to past and future sea-level rise. Nature **531**, 591–597 (2016) https://doi.org/10.1038/nature17145

[5] Pollard, D., DeConto, R.M., Alley, R.B.: Potential Antarctic Ice Sheet retreat driven by hydrofracturing and ice cliff failure. Earth and Planetary Science Letters **412**, 112–121 (2015) https://doi.org/10.1016/j.epsl.2014.12.035

[6] Levermann, A., Albrecht, T., Winkelmann, R., Martin, M.A., Haseloff, M., Joughin, I.: Kinematic first-order calving law implies potential for abrupt ice-shelf retreat. The Cryosphere **6**(2), 273–286 (2012) https://doi.org/10.5194/tc-6-273-2012

[7] Coulon, V., Bulthuis, K., Whitehouse, P.L., Sun, S., Haubner, K., Zipf, L., Pattyn, F.: Contrasting Response of West and East Antarctic Ice Sheets to Glacial Isostatic Adjustment. Journal of Geophysical Research: Earth Surface **126**(7), 2020–006003 (2021) https://doi.org/10.1029/2020JF006003

[8] Le Meur, E., Huybrechts, P.: A comparison of different ways of dealing with isostasy: examples from modelling the Antarctic ice sheet during the last glacial cycle. Annals of Glaciology **23**, 309–317 (1996) https://doi.org/10.3189/S0260305500013586

[9] Lingle, C.S., Clark, J.A.: A numerical model of interactions between a marine ice sheet and the solid earth: Application to a West Antarctic ice stream. Journal of Geophysical Research: Oceans **90**(C1), 1100–1114 (1985) https://doi.org/10.1029/JC090iC01p01100

[10] Bueler, E., Lingle, C.S., Brown, J.: Fast computation of a viscoelastic deformable Earth model for ice-sheet simulations. Annals of Glaciology **46**, 97–105 (2007) https://doi.org/10.3189/172756407782871567

[11] Pollard, D., DeConto, R.M.: Description of a hybrid ice sheet-shelf model, and application to Antarctica. Geoscientific Model Development **5**(5), 1273–1295 (2012) https://doi.org/10.5194/gmd-5-1273-2012

[12] Bernales, J., Rogozhina, I., Thomas, M.: Melting and freezing under Antarctic ice shelves from a combination of ice-sheet modelling and observations. Journal of Glaciology **63**(240), 731–744 (2017) https://doi.org/10.1017/jog.2017.42

[13] Schoof, C.: Ice sheet grounding line dynamics: Steady states, stability, and hysteresis. Journal of Geophysical Research: Earth Surface **112**(F3) (2007) https://doi.org/10.1029/2006JF000664

[14] Pollard, D., DeConto, R.M.: Improvements in one-dimensional grounding-line parameterizations in an ice-sheet model with lateral variations (PSUICE3D v2.1). Geoscientific Model Development **13**(12), 6481–6500 (2020) https://doi.org/10.5194/gmd-13-6481-2020
